# Integration Through Separation – The Role of Lateral Membrane Segregation in Nutrient Uptake

**DOI:** 10.3389/fcell.2019.00097

**Published:** 2019-06-25

**Authors:** Jon V. Busto, Roland Wedlich-Söldner

**Affiliations:** ^1^Institute of Cell Dynamics and Imaging, University of Münster, Münster, Germany; ^2^Biofisika Institute (CSIC, UPV/EHU) and Department of Biochemistry, University of the Basque Country, Leioa, Spain

**Keywords:** plasma membrane, membrane domains, yeast, nutrient transporter, endocytosis

## Abstract

Nutrient transporters are prominent and ubiquitous components of the plasma membrane in all cell types. Their expression and regulation are tightly linked to the cells’ needs. Environmental factors such as nutrient starvation or osmotic stress prompt an acute remodeling of transporters and the plasma membrane to efficiently maintain homeostasis in cell metabolism. Lateral confinement of nutrient transporters through dynamic segregation within the plasma membrane has recently emerged as an important phenomenon that facilitates spatiotemporal control of nutrient uptake and metabolic regulation. Here, we review recent studies highlighting the mechanisms connecting the function of amino acid permeases with their endocytic turnover and lateral segregation within the plasma membrane. These findings indicate that actively controlled lateral compartmentalization of plasma membrane components constitutes an important level of regulation during acute cellular adaptations.

## Introduction

Cells are constantly exposed to environmental fluctuations and thus require rapid adaptations to sustain proper growth and survival. The plasma membrane (PM) constitutes the primary cell boundary and therefore acts as initial site for stress recognition and signaling. The PM is a highly dynamic structure that is compartmentalized in a complex manner. Various models support the lateral segregation of PM lipids and proteins into distinct domains. The budding yeast *Saccharomyces cerevisiae* represents a promising model system for the study of such segregation. Both lipids and proteins of the yeast PM exhibit unusually slow lateral diffusion, and they form large domains that can be studied by conventional light microscopy (Spira et al., 2012a). This allowed the systematic study of a large set of integral PM proteins that revealed an intricate organization of many overlapping domains into a PM patchwork (Spira et al., 2012b).

A critical environmental stress that cells constantly face is nutrient limitation. Starvation is regulated at the PM through a tight remodeling of nutrient transporters, highly abundant and conserved components of the PM in all cells. Transporter turnover in response to substrate availability is modulated *via* two major mechanisms. On the one hand, increased transcription and PM delivery of transporters occur when nutrients are scarce. TORC1 (target of rapamycin complex 1) acts as a central coordinator between protein synthesis, transporter delivery, and metabolic state (Gonzalez and Hall, 2017). On the other hand, transcriptional repression and ubiquitin-dependent internalization prevent excessive substrate uptake when nutrients are abundant. In yeast, selective ubiquitination and downregulation of PM transporters are driven by the concerted action of the E3 ubiquitin ligase Rsp5 and cargo-selective adaptors of the α-arrestin family (Nikko and Pelham, 2009).

Nutrient transporters at the yeast PM have been shown to segregate into different compartments. Prominent examples are the members of the major facilitator (MFS) and amino acid-polyamine-organocation (APC) superfamilies of secondary carriers (Reddy et al., 2012; Vastermark et al., 2014). Various MFS transporters, including hexose and polyamine transporters, distribute into dense, network-like patterns (Spira et al., 2012b). On the other hand, several amino acid and nucleobase transporters of the APC superfamily cluster into defined patches (Malinska et al., 2003, 2004; Grossmann et al., 2007; Bianchi et al., 2018; Busto et al., 2018). Interestingly, all clustered transporters exhibit proton symport activity during substrate uptake. This is particularly striking considering the spatial segregation of the H^+^-symporters from the H^+^-ATPase Pma1 (Malinska et al., 2003). This essential protein drives proton efflux to maintain intracellular pH homeostasis and to generate an electrochemical gradient that provides the basis for metabolite uptake into cells. The segregation of proton-based activities has led to the suggestion that membrane potential regulates lateral distribution of proteins and lipids within the yeast PM (Grossmann et al., 2007; Herman et al., 2015; Malinsky et al., 2016).

Understanding the biological implications behind distinct PM distributions of nutrient transporters and the connection between their function, turnover, and PM compartmentalization has become of broad interest. In this review, we address recent studies on yeast amino acid permeases that reveal their dynamic segregation across different domains in response to substrate availability. These studies show that lateral segregation of transporters can provide protection from endocytic turnover. They also reveal that PM distribution and turnover are regulated *via* a combination of specific protein–lipid interactions and changes in protein conformation that occur during substrate transport. The emerging picture is that lateral PM segregation provides an important and efficient regulatory level to ensure coordination of biological function and turnover for a multitude of nutrient transporters.

## Nutrient Transporter Distribution at the Plasma Membrane

Membrane transporters are ubiquitous components of cell membranes and pivotal in supporting proper cell growth, differentiation, and survival. Transporters can be classified into three main classes: ion channels, primary transporters that mostly use energy in the form of ATP (e.g., ABC transporters, ATPases), and secondary transporters that use an electrochemical gradient to transport a large variety of ions, nutrients (e.g., sugars, amino acids, vitamins, nucleobases, peptides), or small molecules (Saier et al., 2016). The majority of nutrient transporters are expressed at the PM, and their expression levels are tightly regulated by substrate availability. The mechanisms behind expression, PM delivery, and endocytic turnover of nutrient transporters have extensively been studied in budding yeast (Lin et al., 2008; Nikko and Pelham, 2009; Loewith and Hall, 2011; Gonzalez and Hall, 2017). However, detailed knowledge on their dynamic lateral segregation within the PM has only recently emerged.

In a systematic study, Spira et al. (2012b) described the complex distribution of yeast PM proteins into numerous overlapping but distinct patterns that ranged from branched or network-like distributions to patches or clusters. The patterns formed by 46 different proteins were resolved using a combination of TIRF microscopy and 2D deconvolution. The protein set included various nutrient transporters such as the hexose uniporters Hxt1, 2, 3, 6, and the polyamine H^+^-antiporter Tpo1 that all distributed into network-like patterns. These MFS transporters contain 12 transmembrane domains (TMDs) and are structurally organized as two repeat units of six TMDs (Reddy et al., 2012).

**FIGURE 1:**
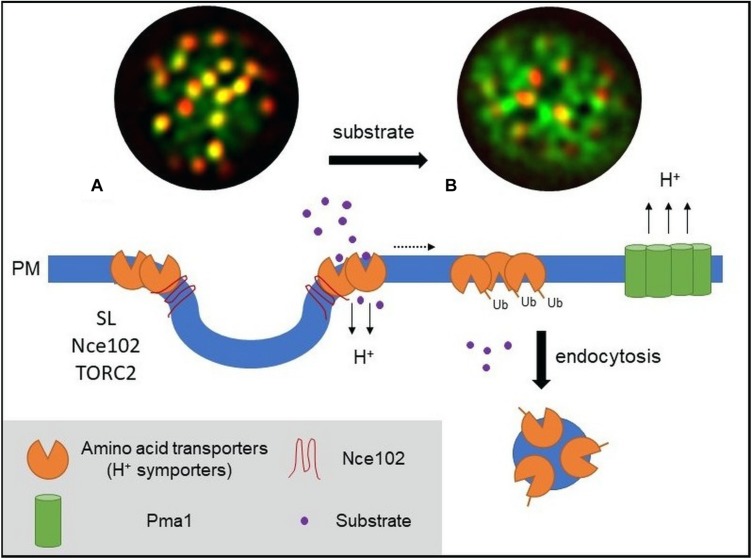
Model of PM amino acid transporter regulation in yeast. **(A)** Amino acid transporters, generally proton symporters, accumulate at the membrane compartment occupied by Can1 (MCC) during substrate starvation. The MCC represents the edge area of PM furrows (also termed eisosome). Clustering of permeases depends on sphingolipids (SL), the tetraspan protein Nce102, and TORC2 signaling. Clustered transporters adopt an open (outward facing) conformation. **(B)** Substrate transport triggers a conformational switch to a closed (inward facing) conformation. This switch is linked to lateral relocation of the transporters out of the MCC (dotted arrow) into a unique PM area that is distinct from that occupied by the H^+^-ATPase Pma1 (MCP). The conformational switch and lateral relocation precede transporter ubiquitination. Ubiquitinated transporters then act as a molecular beacon for the recruitment of the endocytic machinery. Fluorescent images (TIRF) have been adapted from Busto et al. (2018) and represent composites of a ubiquitination-deficient mutant of the methionine permease Mup1 (Mup1-2KR, green) and the eisosomal core component Pil1 (red). Mup1-2KR clusters within the MCC in the absence of methionine (left, colocalization with Pil1 in yellow patches) and relocates out of MCC clusters upon substrate addition.

The yeast arginine permease Can1 was the first permease shown to cluster within a specific compartment at the PM (Malinska et al., 2003), the correspondingly named “membrane compartment occupied by Can1” or MCC. The uracil permease Fur4 (Malinska et al., 2004), the tryptophan and tyrosine permease Tat2 (Grossmann et al., 2007), the lysine permease Lyp1 (Bianchi et al., 2018), and the methionine permease Mup1 (Busto et al., 2018) have been shown to also cluster within the MCC compartment. All five transporters are proton symporters, belong to the APC superfamily, contain at least 12 TMDs, and follow a 5 + 5-fold structural organization with two inverted repeats of five TMDs (Vastermark et al., 2014). Aside from nutrient transporters, the MCC contains various tetraspan proteins and surrounds membrane invaginations called eisosomes that are stabilized by the cytosolic proteins Lsp1 and Pil1. The MCC/eisosome domain has been shown to play a role in sphingolipid (SL) homeostasis, cell wall morphogenesis, and, most relevant for the present discussion, PM organization. Recent studies using super-resolution microscopy confirmed the partition of Can1 and Lyp1 into the outer edge of the MCC/eisosome (Bianchi et al., 2018). A similar observation was made for the methionine permease Mup1 (Figure 1) (Busto et al., 2018).

But what drives particular nutrient transporters to cluster within a specific PM compartment? Previous and recent data converge in showing that clustering is driven by a combination of two main factors: substrate-dependent changes in the conformation of transporters and weak interactions with various proteins and lipids within the established PM domain.

## Dynamic Lateral Relocation Through a Conformational Switch

Endocytic events at the yeast PM have been shown to be excluded from the MCC/eisosome compartment (Grossmann et al., 2008; Brach et al., 2011). As many transporters that cluster in the MCC undergo substrate-dependent endocytosis (Nikko and Pelham, 2009), this would suggest prior relocation out of the MCC. Two independent studies on different nutrient transporters have recently demonstrated this behavior and shed light on the mechanisms underlying such controlled lateral relocation (Busto et al., 2018; Gournas et al., 2018). Busto et al. (2018) showed that, upon substrate uptake, the methionine permease Mup1 undergoes rapid lateral relocation from the MCC into a unique network-like domain at the PM. Lateral relocation occurred rapidly after methionine transport and was independent of ubiquitination and reversible upon substrate washout (Busto et al., 2018). In a remarkable parallel, Gournas et al. (2018) described a nearly identical mechanism for Can1 upon control of the arginine supply. Both studies conclude that a change in the conformational state of the transporters directly controls their lateral segregation within the PM. In the absence of substrate, the transporters are stabilized in an open or outward-facing (OF) conformation that favors MCC association. During substrate transport the permeases shift to a closed or inward-facing (IF) conformation, which drives their lateral relocation out of the clusters (see Figure 1).

More specifically, Gournas et al. (2018) showed that the Can1 S176N mutant, which binds arginine but is locked in the OF conformation (Ghaddar et al., 2014), remains clustered upon substrate addition. In contrast, the inactive Can1 E184Q mutant that is stabilized in the IF conformation is no longer able to cluster in the MCC, even in substrate-free media (Gournas et al., 2018). The case of Mup1 is of particular interest as it is the only yeast amino acid permease that belongs to the L-type amino acid transporters (LAT), a subgroup within the APC superfamily of transporters that has a large number of mammalian members, including the tumor-associated LAT1 (Zhao et al., 2015). One notable feature is a loop in the cytosolic carboxy-terminus of Mup1. Its predicted structure resembles that of a “plug” in the bacterial glutamate-GABA antiporter GadC. Surprisingly, this plug has been shown to insert into the core of GadC and to facilitate a conformational transition during substrate transport (Ma et al., 2012). Consistently, truncation of the C-terminal plug in Mup1 led to Mup1 stabilization within MCC clusters regardless of substrate presence. In contrast, mutation of a conserved motif within the tip of the plug abolished any detectable clustering. Thus, the data clearly support a link between lateral PM segregation of Mup1 and its conformational state, with a key role for the C-terminal plug during the proposed structural switch.

Interestingly, the structural transition to an IF conformation has been proposed to also mediate release of the cytosolic amino-termini, which is crucial for transporter ubiquitination. Recent studies have shown that displacement of the N-terminus in Can1 (Gournas et al., 2017) and Mup1 (Guiney et al., 2016) unmasks PM proximal sequences that are recognized by the α-arrestin Art1 in a first step of transporter ubiquitination and subsequent internalization. However, proper Art1 binding to Mup1 has been shown to require additional recognition sites (Guiney et al., 2016). The C-terminal plug may constitute that additional site. This would then suggest an active role of both cytosolic termini in the conformational switch and in lateral relocation of Mup1. Similar structural rearrangements of the C-terminus upon substrate uptake have been proposed for the abovementioned GadC (Ma et al., 2012) and for the bacterial proton-coupled xylose permease XylE (Wisedchaisri et al., 2014). In addition, C-terminal motifs have been documented to influence transporter activity and structure in Hup1, a heterologously expressed hexose/H^+^-symporter from the green alga *Chlorella kessleri* (Grassl et al., 2000). These findings suggest that common mechanisms and principles might apply to the regulation of transporters in various cells and organisms.

A distinct aspect connecting protein conformation and lateral transporter segregation has recently been put forward (Moharir et al., 2018). Most nutrient transporters that concentrate within MCC clusters are not fully restricted to this domain but can also be observed to various degrees in dispersed networks (Busto et al., 2018; Gournas et al., 2018; Moharir et al., 2018). This suggests a dynamic equilibrium between clustered and non-clustered transporter pools (Brach et al., 2011; Busto et al., 2018; Gournas et al., 2018; Moharir et al., 2018). For the uracil transporter Fur4, it was proposed that the clustered pool within the MCC is kept inactive due to restricted movement in the dense MCC environment. When substrate becomes available, only the non-clustered Fur4 pool would be able to undergo the conformational changes required for substrate uptake. The conformational switch then, in turn, would prevent the transporter from entering the MCC, shifting the equilibrium to the non-clustered PM area. This hypothesis partially contradicts the results with Can1 and Mup1 (Busto et al., 2018; Gournas et al., 2018), which show fully functional uptake of methionine and arginine by transporters that were forced to localize to the MCC domain. Nonetheless, the three studies jointly highlight an active role of conformational states for transporter clustering.

## Cellular Regulation of Nutrient Transporter Clustering

Lateral segregation of nutrient transporters not only depends on conformational changes during the transport process but also is tightly linked to the local PM composition. One prominent factor regulating MCC integrity is the lipid composition of the PM, in particular SLs. SL homeostasis has been proposed to affect MCC composition (Frohlich et al., 2009). Complete inhibition of SL synthesis and selective depletion of complex SLs abolishes Mup1 and Can1 clustering. Both permeases dissociate from the MCC during SL stress. This reorganization is independent of substrate binding and restored by recovery of SL levels (Busto et al., 2018; Gournas et al., 2018). In addition, interfering with yeast sterol biosynthesis also led to reduced clustering of the plant hexose/H^+^-symporter Hup1 within the MCC (Grossmann et al., 2006). In a separate study, the same authors showed that MCC integrity was regulated by the PM-resident tetraspan protein Nce102 (Grossmann et al., 2008), with deletion of NCE102 leading to reduced Can1 and Fur4 clustering. Nce102 was later found to act as a general sensor for SL stress and to regulate Pil1 phosphorylation and stability *via* modulation of Pkh kinase activity (Frohlich et al., 2009). Importantly, a more direct role for Nce102 in clustering of Mup1 and Can1 has been recently advanced. In conditions where Pil1 phosphorylation and, therefore, MCC/eisosome disassembly were prevented, deletion of NCE102 still led to a reduction in permease clustering (Busto et al., 2018; Gournas et al., 2018). This effect was much more prominent for Mup1, which also exhibits much stronger enrichment in MCC clusters to begin with (Busto et al., 2018). Importantly, both studies showed that dispersion of transporters upon SL inhibition occurred even in cells where Nce102 was anchored to MCC clusters either artificially (Gournas et al., 2018) or because it was unaffected by SL stress in methionine-free medium (Busto et al., 2018). The results therefore indicate that lateral segregation of permeases requires a combination of Nce102 and correct lipid composition in the MCC domain.

Signaling through the target of rapamycin complex 2 (TORC2) has been clearly linked to PM organization (Eltschinger and Loewith, 2016). TORC2 localizes to the PM as defined static patches that are segregated from other described PM domains (Berchtold and Walther, 2009). It coordinates actin organization and endocytosis (Rispal et al., 2015) and is a central regulator of SL homeostasis (Berchtold et al., 2012). The recent description of a rapamycin-sensitive TORC2 mutant (Gaubitz et al., 2015) provides an important tool to specifically study its role in PM organization. Noticeably, clustering of Mup1 was regulated by TORC2, as acute TORC2 inhibition by rapamycin led to a strong and rapid reduction in Mup1 clustering (Busto et al., 2018). Concomitantly, an almost complete Nce102 dispersion from the MCC was observed. TORC2 might therefore constitute a central regulator for permease segregation in the PM by simultaneously regulating distribution of tetraspan proteins such as Nce102 and maintaining proper SL homeostasis.

## A Link Between Plasma Membrane Segregation and the Membrane Potential

Under low substrate availability, various proton symporters concentrate on static MCC clusters at the yeast PM. These clusters are spatially segregated from the H^+^-ATPase Pma1, which couples ATP hydrolysis to proton efflux. Using a variety of approaches to perturb membrane potential and the PM proton gradient, Grossmann et al. (2007) demonstrated that the proton symporters, but not the structural MCC resident Sur7, reversibly relocate out of the MCC in response to membrane depolarization. This was shown for Can1, Fur4, Tat2, and, most interestingly, the heterologously expressed Hup1. The authors then proposed that clustering of proton symporters could directly be linked to the activity of Pma1 and highlighted a role of the membrane potential in the lateral compartmentalization of PM domains. Hup1 belongs to the MFS superfamily, while yeast permeases are APC transporters. The common denominator for MCC clustering and membrane potential-mediated relocalization seems to therefore not reside within the intrinsic transporter structure but rather in their proton symport activity.

Remarkably, forced relocation of Mup1 and Can1 from MCC clusters to the Pma1-enriched compartment strongly inhibited the respective substrate uptake (Spira et al., 2012b; Busto et al., 2018). In contrast, MCC disassembly upon depletion of Pil1 with subsequent loss of permease clustering did not affect the transport activity (Busto et al., 2018; Gournas et al., 2018). This suggests that the Pma1 domain has an inhibitory effect on symporter activity, rather than the MCC environment having a stimulatory function. Together, the data suggest that the Pma1 domain constitutes a physicochemical environment that is harmful for proton symporters and that lateral segregation may be an efficient way to separate the generation and utilization of the electrochemical gradient across the PM (Figure 1). Future analysis in different cellular systems will have to show whether this is a fundamental feature that also holds true for proton gradients in plants or Na^+^–K^+^ gradients in animal cells.

## Interplay Between Lateral Segregation of Nutrient Transporters and Endocytic Turnover

Lateral confinement of nutrient transporters and specifically of proton symporters in yeast not only impacts nutrient uptake but also is intimately linked to endocytic recycling and degradation of the transporters. A physiological role of the MCC as protective area from endocytosis was proposed earlier (Grossmann et al., 2008). However, while the absence of endocytosis within the MCC was later confirmed (Brach et al., 2011), the remaining pool of MCC-associated Can1 after substrate addition questioned an active role of the MCC in endocytic regulation (Brach et al., 2011). The recent studies add further details to the consequences of transporter clustering on endocytic recycling. For both Mup1 and Can1, clustering and substrate-induced lateral relocation are ubiquitination-independent processes (Busto et al., 2018; Gournas et al., 2018). Importantly, forced retention of the transporters within the MCC patches does not prevent substrate uptake – i.e., the required conformational shifts – but completely precludes transporter endocytosis. In addition, ubiquitination of Can1 was already blocked when the transporter was physically tethered to the MCC *via* an artificial anchor (Gournas et al., 2018). MCC disassembly restored Mup1 and Can1 endocytosis, including that of a constitutively clustered Mup1-ubiquitin fusion. The results therefore indicate that clustering of transporters in the MCC compartment provides protection from both ubiquitination and endocytosis.

Why would a cell need areas on the PM where proteins cannot be internalized? Possible physiological scenarios where this becomes highly relevant are systematic cell stress (temperature, salt) or acute starvation. In these conditions, cells will actively remove proteins and lipids from the PM to gain metabolic building blocks and reduce energy consumption (particularly by the H^+^ pump). Such a global downregulation can occur by autophagy (Boya et al., 2013) or widespread ubiquitin-mediated endocytosis (Muller et al., 2015; Huber and Teis, 2016). Importantly, cells still need to be able to sense nutrients even in an adapted or depleted state. Therefore, a small population of nutrient transporters retained in the PM would pose a significant advantage. Exactly such a role for the MCC in providing a safe haven under acute starvation conditions has been proposed for Can1 (Gournas et al., 2018). The authors showed that upon general amino acid starvation and in stationary phase, the number and surface area of MCC clusters were increased, providing a protected reservoir for transporters during a global TORC-mediated removal of PM proteins.

While clustering of transporters is an efficient way to regulate global turnover, it is not sufficient to explain the metabolic fine-tuning that cells can perform in response to availability fluctuations of individual substrates. Clustering is a dynamic process and there will always be a large portion of PM proteins outside of the MCC domain. An important question for specific regulation of transporter abundance is therefore how internalization can be focused on one particular cargo. The recent finding of substrate-induced assembly of endocytic structures (Busto et al., 2018) could provide the required link. It was shown that ubiquitinated Mup1, once relocated outside of the MCC, acts as a molecular beacon for recruitment of the endocytic machinery and subsequent internalization. These endocytic sites are clearly segregated from the area containing Pma1 and likely also do not include unrelated transporters that are partially present in the internalized PM region (Figure 1). The combination of conformation-mediated lateral relocation and cargo-dependent assembly of endocytic coats could provide a good pathway for specific adjustment of PM composition and cellular metabolic activity.

The recent studies on turnover of yeast nutrient transporters provide general insights into a role of lateral PM segregation for compartmentalization of biological functions. However, the molecular details of the PM regulation of nutrient transporters are far from being fully understood. Future experiments attempting to directly visualize PM lipid distribution during transporter activation and lateral relocation could help to better assess the nature of the molecular interactions behind transporter segregation. Such experiments will help to untangle the respective roles of protein–lipid vs. protein–protein interactions for dynamic cluster formation. Furthermore, isolation and quantitative analysis of endocytic vesicles during substrate-induced transporter turnover will provide a more comprehensive picture of the molecular composition of endocytic PM areas.

## Author Contributions

RW-S and JVB wrote the manuscript and coordinated the project.

## Conflict of Interest Statement

The authors declare that the research was conducted in the absence of any commercial or financial relationships that could be construed as a potential conflict of interest.
